# Evolution Decision, Drivers and Green Innovation Performance for Collaborative Innovation Center of Ecological Building Materials and Environmental Protection Equipment in Jiangsu Province of China

**DOI:** 10.3390/ijerph15112365

**Published:** 2018-10-25

**Authors:** Wei Fang, Lulu Tang, Pengxiao Cheng, Naveed Ahmad

**Affiliations:** School of Management, Northwestern Polytechnical University, Xi’an 710072, China; tanglulu@mail.nwpu.edu.cn (L.T.); chengpengxiao@mail.nwpu.edu.cn (P.C.); naveedahmad@mail.nwpu.edu.cn (N.A.)

**Keywords:** collaborative innovation, green innovation, driver, qualitative simulation, analytic network process, decision

## Abstract

Faced with the bottlenecks and shortcomings brought about by the resource and environmental issues regarding the sustainable development of the economy and society, green innovation has become an important symbol to measure the sustainable competitive advantage of a country and a region. As an important carrier of green innovation, the evolution process of the collaborative innovation network and its green innovation performance are affected by many factors. Therefore, this paper refines the influencing factors of the formation and evolution of collaborative innovation networks and the evaluation indicators of the green innovation performance by literature analysis. According to the characteristics of each evolutionary influence factor, the relationship governance mechanism, relationship strength, and dominant role are defined as decision factors. The rest are defined as drivers. Then, the Analytic Network Process (ANP) is used to empirically analyze the interaction between network evolution decision, driving factors, and green innovation performance, and the interaction relationship model of decision factors, driving factors, and green innovation performance is obtained. The qualitative simulation algorithm based on qualitative simulation (QSIM) basic theory is used to simulate the evolution of a collaborative innovation network, and find the optimal decision to make the green innovation performance reach its relatively high point. Finally, this paper considers the Collaborative Innovation Center of Ecological Building Materials and Environmental Protection Equipment in Jiangsu Province of China as the research object, focusing on its initial stage of growth and maturity. Combining the theory of QSIM with the actual simulation, according to the different development stages of the Collaborative Innovation Center, this paper provides decisions that can promote the rapid improvement of green innovation performance in three aspects: relationship governance mechanism, relationship strength, and core leadership.

## 1. Introduction

After Chinese economic reform and the subsequent open-door policy, China’s science and technology have undergone significant growth and improvement. However, in the face of global economic development, China has entered a new round of economic transformation; meanwhile, innovation has become a crucial factor affecting the rapid development of the economy. Collaboration can increase innovative capability and generate creative outputs [1]. Collaboration refers to activities where two or more business partners contribute their resources and capabilities to achieve common objectives. Strong collaboration and enhanced innovation capability lead to the synergy that allows an organization to achieve together with other organizations things that could not be achieved alone. The concept of synergy was firstly introduced by Ansoff (1987) [2] in the book ”Corporate Strategy” in 1965. In the 1970s, the German physicist Haken (1984) [3] firstly systematically proposed synergy theory and integrated the concept of synergy into an organizational context. He believed that synergy referred to the synergistic behavior of subsystems that produces a separate effect beyond the elements themselves in a complex large system, thus forming a joint role of the entire system. In the evolution of analyzing complex systems, Corning (1983) [4] defined synergy as ”the combined effect of two or more subsystems, elements, or people in a natural or social system through interdependence.” Tidd et al. (2013) [5] applied the synergy theory to the field of technological innovation and conducted in-depth research on the synergy of various innovation elements in the enterprise at the micro level, which enabled the synergy and innovation concept to achieve effective integration. Collaborative innovation is a systematic innovation that has the natural attributes of networking. The academic community proposes the concept of a collaborative innovation network based on collaborative innovation research. According to the definitions from Baba et al. (1989) [6] and Freeman (1991) [7], the collaborative innovation network is a cross-border organization, which is a basic institutional arrangement for dealing with systemic innovation. The innovative collaborative relationship between innovation subjects is its main link mechanism. Some scholars take the network perspective and use social network analysis to study the changes to network structures in the process of collaborative innovation network evolution to explore the network structure that can enhance innovation performance [8,9]. Some scholars have also studied the specific influencing factors on the innovation performance improvement process of a collaborative innovation network [10,11]. Although there are certain studies on the influencing factors, there is still a lack of systematic research on the influencing factors at the theoretical level.

However, while improving its innovation performance, China also faces the challenge of balancing its future urbanization processes with resource conservation and environmental protection. The Chinese government believes that promoting sustainable development is an important strategy to address these challenges. To promote sustainable construction, the Chinese government has introduced a large number of laws, policies and regulations [12]. In response to government calls to ease environmental pressures, many companies have begun to integrate environmental protection and environmental management into their management agendas [13]. Scholars believe that green innovation is not only a general innovation activity, but also has the characteristics of achieving resource conservation and environmental improvement. Therefore, this paper defines green innovation as a resource-saving, environment-friendly, and sustainable innovation. For enterprises, some scholars have proposed that green innovation activities such as producing green products and upgrading green manufacturing technologies contribute to environmental protection, and can also enhance the core competitiveness of enterprises and improve the ecological environment [14]. Some scholars also study green innovation from the aspects of green product innovation and green process innovation [15]. Therefore, improving green innovation performance has become an important path for enterprise development.

In the process of economic development, green innovation is one of the important measures to protect the environment. Therefore, this paper takes the collaborative innovation network as the main body, and studies and analyzes all of the factors affecting its green innovation performance. Based on the characteristics of all of the factors, they are divided into two categories: driving factors and decision factors. Then, this paper uses the Analytic Network Process (ANP) to explore the interaction between all of the factors. This paper builds a simulation model based on the improved algorithm of qualitative simulation theory. Simulation is performed by MATLAB software. The simulation results are finally analyzed, and management suggestions are provided.

From a theoretical perspective, this paper systematically summarizes the influencing factors affecting the green innovation performance of collaborative innovation networks through the combing and analysis of previous literature material and actual research. All of the influencing factors are classified into specific categories, which are mainly divided into two major categories: driving factors and decision factors.

This paper is structured as follows. Section 2 reviews the collaborative innovation and green innovation performance theory, and refines the influencing factors of green innovation performance. Section 3 establishes a correlation model of all of the influencing factors, and also establishes a qualitative simulation model. Section 4 is based on the analysis of collaborative innovation, and proposes management implications based on the results. Section 5 provides the main summary.

## 2. Theoretical Background

The collaborative innovation network has complex system characteristics and is a complex adaptive network system. Hadjimanolis (1999) [16] pointed out that a collaborative innovation network is composed of nodes that are formed between various innovation entities that are either vertical or horizontal. Maietta (2015) [17] has divided the industry from the perspective of industry technology, focusing on the field of traditional low-tech industries, and exploring the elements of the collaborative innovation network that affect the innovation effect of low-tech industries. Nowadays, the main institutions, such as enterprises, universities, research centers, government, banks, and intermediaries are generally included in the collaborative innovation network. Due to the constant change caused by the complexity of the innovation network, we need to explore it in depth and study its development rules in order to enhance its innovation performance.

Green innovation performance is explored from different angles. Some scholars consider it from government agencies. Hepburn Cameron et al. (2018) [18] believed that policy funding has a significant positive impact on environmental innovation initiatives. Qi et al. (2010) [19] pointed out that environmental management issues and the environmental regulations of enterprises in the construction industry have had a significant effect on the green behavior of enterprises, and the pressure of stakeholders has had no significant influence on green behavior. Some scholars have considered it from the perspective of knowledge and technology. Lee et al. (2011) [20] studied the improvement of aircraft fuel efficiency over the past 40 years, and believed that environmental pollution, low-resource utilization, and technological innovation are the main predictors of green innovation performance. Albort-Morant et al. (2018) [21] studied the impact of corporate absorptive capacity on green innovation performance by analyzing the entire stages of acquiring, absorbing, transforming, and utilizing external environmental knowledge. This research showed that knowledge transfer activities have had a strong role in promoting the green innovation performance of enterprises. Some scholars have considered these networks from the perspective of the communication process. Horowitz et al. (2017) [22] believed that communication, collaboration, and common willingness contribute to the improvement of innovation efficiency in a community network with more entities. Du et al. (2017) [23] found that the fairness of the distribution of benefits had a significant impact on the change in innovation performance. Robin’s research on 202 Taiwanese service and manufacturing companies found that competitors and government pressures had a positive impact on corporate green innovation performance [24]. Some scholars also considered it from the perspective of business and management. Triguero et al. (2013) [25] surveyed European small to medium enterprises (SMEs), and found that green product demand and production costs had a positive effect on green innovation. Moreover, existing regulations have had an impact on innovation and expected regulations, and subsidies have not had an effective impact. Robertson [26] proposed that the company’s environmental protection behavior is mainly reflected in the employee’s voluntary approach to environmental protection, and the main factor that affects employee behavior is whether the leader has environmental protection awareness or not. Thus, a leader’s environmental protection awareness has a significant influence on a company’s overall green innovation activities.

The influencing factors mentioned above are mainly studied in three aspects: the environment in which the network is located, the attributes of the main body in the network, and the internal mechanism of the network. It can be found that these factors are not allowed to be directly changed by humans, but can be judged by surveys and observations. Therefore, this paper defines them as driving factors.

In the process of developing collaborative innovation networks, the innovation network is influenced by a governance mechanism, which indicates the relationship strength between innovation entities and the innovation network, and is dominated by different attribute entities at different stages. Liu et al. (2016) [27] believed that the relationship governance mechanism can be divided into two types: trust and contract control. Through these two methods, the cooperating entities can have different perceptions of fairness, which affect the long-term cooperation and knowledge transfer efficiency. In terms of the strength of the relationship, it is generally divided into a strong connection and a weak one, and both of them present positive promotion to cooperation. However, strong connections generally lead to information redundancy, while weak links promote the transmission of new knowledge content [28]. At the same time, enhancing or weakening the relationship strength with the partners will affect the main knowledge content and the efficiency of knowledge transfer to varying degrees. It will affect the communication coordination and benefit the distribution mechanism within the entire network to ultimately affect the value and performance of the entire cooperation [29]. In the collaborative innovation network, the main players in the core positions are more likely to gain the connection advantages. Their overall strength becomes stronger and stronger, and they will have a significant impact on the resource allocation and innovation output in the network [30]. From the above-mentioned literature, we can find that the relationship governance mechanism, relationship strength, and core leadership are the main parts promoting the change of related drivers, and the change of driving factors leads to changes in the innovation performance. Therefore, we believe that there is an indirect relationship between relationship governance mechanisms, relationship strength, core leadership, and innovation performance. Changing them to different collaborative innovation network states can lead to changes in driving factors, which in turn effect changes in innovation performance. Therefore, this article defines them as decision factors. Since the collaborative innovation network is a complex and evolving network, the driving factors have different states in different stages of evolution, and the green innovation performance is also changing. Therefore, when the performance of green innovation is no longer growing at a certain stage, the internal entities of the network need to change the decision factors, so that the driving factors will constantly change through mutual influence, and then the green innovation performance will be improved again. When the performance of green innovation changes, the degree of change will also have a feedback effect on the driving factors, and the driving factors will change again. The influence of this kind of non-linear superposition has always been accompanied by the development of collaborative innovation networks. It continuously improves the output of green innovation. The specific sources of all of the factors are shown in Table 1.

Through the above analysis, this paper finally divides all of the factors in the collaborative innovation network into three categories: performance, drivers, and decision factors. The driving factors include the attribute factors, regional macro factors, regional micro factors, and communication factors. Decision factors include the relationship governance mechanism, relationship strength, and core leadership. All of the factors are classified as shown in Table 2.

In summary, there is a mutual influence between the evolutionary decision, driving factors, and green innovation performance of collaborative innovation networks. In order to explore the optimal promotion strategy of the collaborative innovation network’s green innovation performance, this paper uses ANP to conduct empirical research to analyze the multi-level complex dynamic relationship among various factors in the network. On this basis, through the improved qualitative simulation (QSIM) algorithm, the dynamic qualitative simulation of the relevant factors of the collaborative innovation network is carried out.

## 3. Method

### 3.1. ANP and Impact Weight Analysis

The Analytic Network Process is a new decision-making method proposed by Professor Saaty in 1996 that is based on the analytic hierarchy process. It is an extension of the analytic hierarchy process. The characteristic of ANP is that it fully considers the interaction between elements or adjacent levels on the basis of the analytic hierarchy process. Through direct dominance and indirect dominance, a comparison matrix between the elements is constructed, and a “super matrix” is used to comprehensively analyze the elements of the interaction relationship to obtain the mixed weight. The analytic network model does not require the complex hierarchical relationships that the analytic model does, and there may be interactions between decision layers or within the same layer [48]. Therefore, ANP is suitable for studying the relationships between all of the factors of the collaborative innovation network in this paper, and analyzing their impact on network evolution and innovation performance. A step-by-step approach to apply ANP for checking the mutual influence between evolutionary decisions, driving factors, and green innovation performance is described below:

(1) Obtaining Relevant Factors

This study uses questionnaires to obtain and collect data. The questionnaire was designed to investigate the degree of influence between all of the factors. The score was judged on a scale of 0 to 10. The score gradually increased according to the degree of influence, where 0 means no effect, and 10 indicates an absolute impact. This paper selects experts and scholars from enterprises, universities, research institutes, and technology intermediaries included in the typical collaborative innovation center. A total of 50 questionnaires were distributed, and 50 questionnaires were finally collected. The response rate was 100%. According to the identity of the respondents, there were 20 senior technical personnel and corporate executives, accounting for 40%; 17 “Double First-Class” college professors, accounting for 35%; and 13 researchers from research institutes and related intermediaries, accounting for 25%. After discussions, the relevant data was finally obtained. The experts and scholars who were selected in this paper are professionals who have worked in related professions and positions for more than 20 years. They include senior technical experts, senior management, etc. They have sufficient theoretical research and practical experience in green innovation technology and management. Finally, by statistical analysis, the average degree of influence between all of the factors is obtained. The influence degree that each factor receives from related elements can also be acknowledged.

(2) Building a judgment matrix

According to the degree of relevant influence of all of the factors and the magnitude of the influence degree, the indirect dominance comparison is performed on a 1–9 scale, where one point indicates the slightest impact, and nine points indicates the strongest impact. In this paper, the scales *c_i__,j_* between the element groups *L_i_* and *L_j_* are calculated. The judgment matrix *C* between the element groups is obtained by normalization processing. The scale *b_i__,j_* is obtained from the score between the elements *I_i_* and *I_j_* in the element group *L_i_* and *L_j_*. The judgment matrix *B_i__,j_*, which gives the importance of the influence of the group *L_j_* on the elements in the group *L_i_*, is obtained by normalization processing. *B*_2_*_,_*_3_ indicates the importance of the influence of the four elements in the element group *L*_2_ on the four elements in the element group *L*_3_. *B*_2,6_ indicates the importance scale of the influence of element *I*_2_ on element *I*_6_. Until each element group and all of the element groups successfully construct the judgment matrix *B_i,j_*, all of the judgment matrices *B_i,j_* between the indicated elements are integrated according to the position corresponding to the judgment matrix *C*. Finally, the super matrix *B* is obtained.

(1) $$C=\left(\begin{array}{ccc} c_{1,1} & \ldots & c_{1,8}\\ \vdots & \ddots & \vdots \\ c_{8,1} & \cdots & c_{8,8} \end{array}\right)\;$$

(2) $$B_{2,3}=\left(\begin{array}{ccc} b_{2,7} & \ldots & b_{6,7}\\ \vdots & \ddots & \vdots \\ b_{2,10} & \cdots & b_{6,10} \end{array}\right)\;$$

(3) $$B=\left(\begin{array}{ccc} B_{1,1} & \ldots & B_{1,7}\\ \vdots & \ddots & \vdots \\ B_{7,1} & \cdots & B_{7,7} \end{array}\right)=\left(\begin{array}{ccc} b_{1,1} & \ldots & b_{1,n}\\ \vdots & \ddots & \vdots \\ b_{m,1} & \cdots & b_{m,n} \end{array}\right)\;$$

(3) Establishing a weighted super matrix

Equation (4) indicates that the transposed matrix *C^T^* of the judgment matrix *C* is multiplied by the super matrix *B.* The weighted super matrix *W* is obtained by column normalization.

(4) $$W=C^{T}\times B\;$$

(4) Calculating the limit super matrix

The limit super matrix is calculated by Equation (5), which represents the relative ranking weight of the element index:

(5) $$w=\underset{x\to \infty }{\lim}W^{x}\;$$

The final impact factor weights obtained by calculation are shown in Table 3 and Table 4, which show the influence of column elements on row elements.

**Figure 1:**
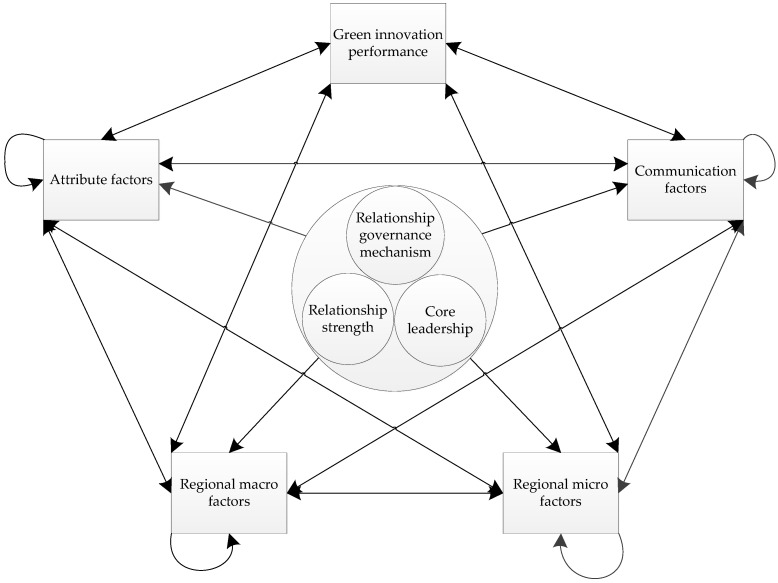
Collaborative innovation network green innovation performance-related factors relationship model.

By affecting the degree value and the relative ranking weight of all of the final element groups and elements, we can get the relationship model of all of the elements, as shown in Figure 1.

### 3.2. Qualitative Simulation

The QSIM algorithm was proposed by Kuipers (1990) [49] from the University of Texas in the United States (USA). He believed that qualitative simulation is a constraint-oriented method that predicts all of the possible future behaviors of the system from a constraint set and an initial state. Specifically, to achieve the qualitative simulation of a certain system, the system structure should be firstly described by using the parameters in the system as state variables. Secondly, the constraint relationship is obtained by the laws of physics, and the change of parameters over time is regarded as the sequence of qualitative states. Finally, starting from the initial state, all of the subsequent states are generated. The wrong system state is excluded based on the physical relationships prior to the system reaching a steady state. In summary, qualitative simulation is the study of system structure, behavior, function, and the relationship and cause and effect between them. Qualitative simulation is a cross-domain method of reasoning, aiming at exploring the common sense mechanism to effectively solve multiple tasks [50].

Qualitative simulation is suitable for objects that cannot be accurately described by mathematical models. In the process of development and the management of a collaborative innovation network, it is very suitable to use the qualitative simulation method to explore, considering that there are many factors and the relationship is complex and not fixed.

However, due to the complex nature of the collaborative innovation network, the QSIM algorithm cannot fully adapt to the interaction between the internal variables of the network. Therefore, with reference to the research of Wei et al. (2013) [51], this paper improves the quantitative level based on the basic theory of the QSIM algorithm. At the same time, according to the results of the ANP method, this paper considers the interactive feedback between the factor variables, using the MATLAB software for qualitative simulation.

#### 3.2.1. Variable Description

According to the empirical research results and the qualitative simulation theory, firstly, all of the influencing factors are defined as state variables. Secondly, according to the relational model obtained from the empirical research, this paper defines the relationship between factors and adds influence weights as path coefficients. There are positive and negative factors in the influence relationship. The weight value of the negative influence relationship is defined as a negative value.

Specific variables are described below:

(1) Network evolution drivers

Describe all of the factor variables using the two-group method: the qualitative state of the variable at time *t* is QS(G,t)=(qval,qdir) $G\in \left\{I_{2},\cdots \cdots I_{18}\right\}$. The *t* is the analog clock. The *qval* is the qualitative state value of the variable qval∈(1,2,3,⋯⋯98,99,100). The variable status is gradually changed from “very low” to “very high”. The *qdir* is the trend of variables, qdir∈[−2,2]. This represents “substantial reduction” to “large increase”. Green innovation performance is defined as the variable *U* in QS(U,t)=(qval,qdir). The qualitative state value of *U* has no upper limit due to the infinite growth of the green innovation performance, qval∈{1,2,3,⋯⋯+∞}. The other variables are represented in the same way as the variable *G*.

(2) Network evolution decision

Decisions are dependent on the selection of variables. In the realistic collaborative innovation network, there is a facility of practicing one choice at a certain time. For example, for a relationship management mechanism, either trust or a contract control decision can be chosen to execute one of the policies at a certain time. It is impossible for the process of policy execution to be described quantitatively. A choice can only be made as to whether to execute the policy or not. So, the state value of the defined policy factor variable is either 0 or 1, where 0 means no selection, and 1 means select execution. This paper uses *X*, *Y*, *Z* to define the relationship governance mechanism, relationship strength, and core leadership.

$$X\in \left\{I_{19},I_{20}\right\}\;Y\in (I_{21},I_{22})\;Z\in (I_{23},I_{24},I_{25})\;$$

#### 3.2.2. Model Optimization

According to the initial state of different collaborative innovation networks, what measures can be taken to optimize the green innovation performance? The collaborative innovation network evolution decision can be transformed into the optimization model as follows. Equation (6) is the objective function, which means that the green innovation performance is maximized under the influence of decision. Equations (7)–(9) represent the number of specific strategies under the three governance categories: relationship governance mechanism, relationship strength, and core leadership. Equations (10) and (11) are qualitative binary representations of green innovation performance and network drivers. Equation (12) shows the interaction between all of the variables. The specific relationship is expressed in Section 3.1 of this paper.

(6) $$MAX\;U\left(I_{1}{,I}_{2}{,I}_{3}{,\mathrm{LI}}_{18},X_{l},Y_{m},Z_{n}\right)\;$$

$$s.t.\left\{ \begin{array}{cc} l\in \left(1,2\right) & \;(7)\\ m\in \left(1,2\right) & \;(8)\\ n\in (1,2,3) & \;(9)\\ U_{i}=(qval_{i},qdir_{i}),i=1 & \;(10)\\ G_{i}=(qval_{i},qdir_{i}),i\in (2,3\cdots 18) & \;(11)\\ F\left(U_{k},G_{i},X_{l},Y_{m},Z_{n}\right)=0 & \;(12) \end{array}\right.$$

#### 3.2.3. Simulation Algorithm

(1) Calculation and conversion rules

This paper defines the calculation and conversion of the state values and trends of variables *U* and *G*. When the decision is implemented, it will affect the status of all of the factors, which will lead to changes regarding the green innovation performance. Therefore, during the simulation algorithm for an initial state, the decision only affects the initial state of the network. At the same time, all of the network drivers, including the increase in green innovation performance, have the characteristic of diminishing marginal effects.

* calculation steps

- At *t*_0_, the decision combination is formed by decision selection. Then, with the selected decision, the influence weight of the relevant network driver and the state value of the factor itself are calculated. The trend value *qdiri*_1_ of the network driving factor at the time *t*_1_ is obtained with the help of Equation (13):
  (13) $$qdir_{i1}=\left(X_{l}*W_{li}+Y_{m}*W_{mi}+Z_{n}*W_{ni}\right)*e^{-\frac{qval_{i1}}{100}}\;$$
- Through the conversion rule, the change value *C_i_*_1_ of all of the state factors affected by the decision at step *t*_1_ is judged. The state value is increased or decreased. All of the network driver factor status values *qvali*_1_ at step *t*_1_ are obtained. According to the change value of all of the factors and the correlation value affecting the weight value, the trend of the network driver in step *t*_2_ is obtained through Equation (14):
  (14) $$qdir_{i2}=e^{-\frac{qval_{i2}}{100}}*\sum_{i=1}^{13}C_{i2}\cdot W_{ij}\;$$
- Through the conversion rule, the change value of all of the network driver factor state values with a mutual influence relationship at step *t*_2_ is judged. The state value is increased or decreased. All of the network driver factor state values at step *t*_2_ are obtained. As the step *t* progresses, the loop repeats steps *c*, *d* until all of the factor state values no longer change.

* Conversion rule

This paper refers to the research of Wei et al. (2013) [51], where a judgement is made based on the factor change trend value *qdir_ik_*, which is calculated at *t_k_* step. The conversion rule is described below:

If *qdir_ik_* > 1, *qval_ik+_*_1 =_ *qval_ik_* + 3, *C_ik+_*_1_ = 3,

If 0.5 < *qdir_ik_* < 1, *qval_ik+_*_1 =_ *qval_ik_* + 2, *C_ik+_*_1_ = 2,

If 0.1 < *qdir_ik_* < 0.5, *qval_ik+_*_1 =_ *qval_ik_* + 1, *C_ik+_*_1_ = 1,

If −0.1 < *qdir_ik_* < 0.1, *qval_ik+_*_1 =_ *qval_ik_*, *C_ik+_*_1_ = 0,

If −0.5 < *qdir_ik_* < −0.1, *qval_ik+_*_1 =_ *qval_ik_* −1, *C_ik+_*_1_ = −1,

If −1 < *qdir_ik_* < −0.5, *qval_ik+_*_1 =_ *qval_ik_* – 2, *C_ik+_*_1_ = −2,

If *qdir_ik_* < –1, *qval_ik+_*_1 =_ *qval_ik_* – 3, *C_ik+_*_1_ = −3,

If *qval_k_ =* 100 and *C_k_* > 0, *qval_k+_*_1_ *=* 100,

If *qval_k_ =* 1 and *C_k_* < 0, *qval_k+_*_1_ *=* 1.

(2) Algorithm step

Use *G*_0_ to indicate a set of initial state values. The initial value of green innovation performance is defined as 0. There are seven strategies. The amount of decision combinations is 12. Define *p* = 1, *k* = 0.

First step: Enter the *p*-th group decision combination and initial state value *G*_0_.

Second step: Start *G* and *U* state value conversion; define *k* = *k* + 1.

Third step: If *QS(G,t_k+_*_1_*) = QS(G,t_k_)* and *QS(U,t_k+_*_1_*) = QS(U,t_k_)*, this means that the value of the entire collaborative innovation network factor has reached a stable level. Green innovation performance no longer grows. The innovation performance value *U* at this time is the final value obtained under the influence of the *p*-group decision combination. If the condition is not true, repeat the second step.

Fourth step: If *p* < P, let *p* = *p* + 1, go to the first step; otherwise, continue.

Fifth step: The optimal value and the optimal decision are obtained by comparing all of the decision combinations with their corresponding final green innovation performance values.

## 4. Case Analysis and Management Implication

According to the characteristics of the QSIM algorithm, this paper defines the initial trend value of all of the factors as zero. To effectively compare the effects of the decision, the initial state value of green innovation performance is defined as one. The applicability of the decision is measured by the value of the green innovation performance indicator when the final system converges. At the same time, this paper selects the Collaborative Innovation Center of Ecological Building Materials and Environmental Protection Equipment in Jiangsu Province of China as the research object. Construction sites are prone to cause pollution sources such as exhaust gas, dust, noise, and construction waste, which seriously affect human ecological environment. Under the guidance of the Yan City People’s Government of Jiangsu Province, the Industry, University, and Research Institute jointly established the Jiangsu Province Ecological Building Materials and Environmental Protection Equipment Collaborative Innovation Center, which focused on green environmental protection innovation. The center aims at solid waste recycling, flue gas purification, and construction site environmental protection equipment to solve some existing pollution problems on the construction site. After the investigation, it was found that the Collaborative Innovation Center of Ecological Building Materials and Environmental Protection Equipment in Jiangsu Province was established in recent years. It was in the early stage of development. Many entities formed a network structure through contact. Therefore, based on the theory of qualitative simulation, we understood the status quo of the influencing factors at the current stage by interviewing experts and scholars who have been working in the Collaborative Innovation Center for a long time. At the same time, we analyzed and evaluated the results of the interviews and obtained appropriate scores. We defined the score as 0 to 100. The specific indication of the score is slightly different for different factors. This score is used as the initial value of the first stage of the simulation. Some factors are in good condition to get a high score. For example, green innovation capabilities, green innovation willingness, etc. Some factors have poor status and get a high score. For example, green technology difficulty, green product cost, etc. Then, the simulation is carried out. Once the first stage of operation is completed, the final state of the previous stage is simulated again as the initial status of the next stage until the system reaches steady state. The final assignment data and simulation results are shown in Table 5.

**Figure 2:**
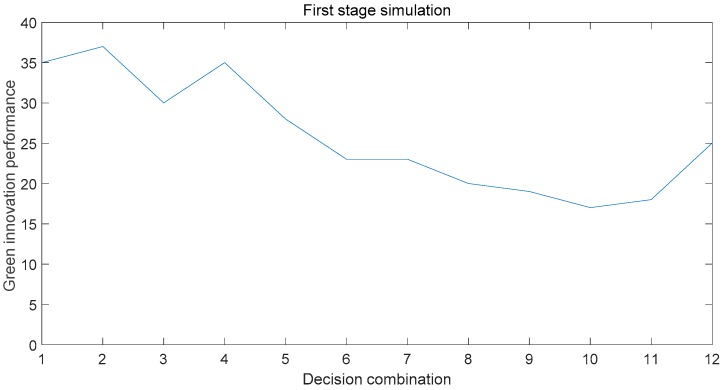
First-stage simulation results.

According to the simulation results of MATLAB, the initial assignment of the first stage has the influence of 12 different selection combinations on the green innovation performance. It is found that the second combination can make the innovation performance reach the optimal value, as shown in Figure 2. The Collaborative Innovation Center is at the initial stage of development. Although China strongly supports green technology innovation, it is more important for the upgrading of environmental pollution control technology based on the ecological environment. In response to the key points of the problem, combined with the needs of stakeholders, we can better solve the problem of people and nature. Therefore, when the Collaborative Innovation Center is established, the center should take the university research as the core. First, to quickly identify the problem and the direction of improvement in the future, the center should explore the key issues of pollution. Then, strong connections should be established between the main bodies to strengthen communication and facilitate the rapid transmission of information. In this way, the realities of all of the aspects of the pollution problem can be grasped with the least cost, so that the problem can be quickly analyzed, and more solutions can be proposed for selection. At the same time, trust is used as the relationship governance mechanism. In the condition of mutual trust, each main body can be made to have a sense of identity and enhance the cohesiveness of the collaborative innovation network.

**Figure 3:**
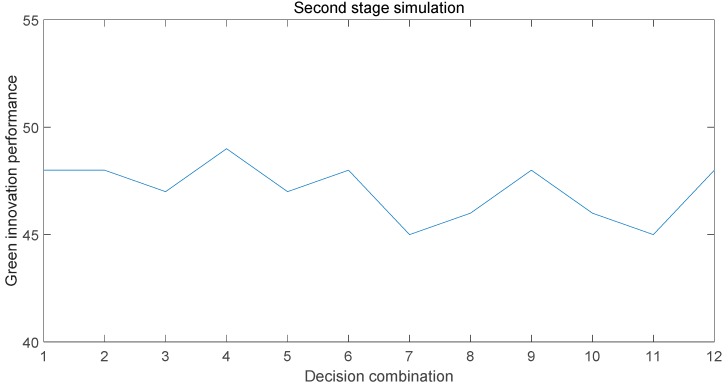
Second-stage simulation results.

After the initial simulation, under the influence of the initial optimal decision combination, all of the relevant factors have reached a stable state. It is difficult for green innovation performance to rise after reaching the optimal value. To further improve the green innovation performance, the value of all of the factors reaching the steady state is again simulated as the initial value of the second stage, as shown in Figure 3. According to the results of the simulation, after the replacement of the decision combination, the green innovation performance is improved again. Selecting the fourth decision combination in the second phase can improve the green innovation performance and make it reach the relative optimal value. Combined with the actual analysis of the simulation results, it can be seen that we should continue to use trust as the basis of relationship governance to enhance the relationship maintenance between the subjects when the development of the Collaborative Innovation Center reaches a certain stage. Gaining trust can induce subjects to feel dependent, which will easily lead to the subject actively carrying out green innovation behavior. Proactive green innovations have a positive impact on green innovation performance compared to being forced to engage in green innovation [52]. After the first phase of the collaborative innovation network, the center will inevitably attract some new subjects to join. Therefore, a weak connection relationship should be established, which can promote the transmission of new ideas, new knowledge, and new information, and reduce the small groups and information redundancy formed by the strong connection. At the same time, the influx of new knowledge will lead to an increase in science and technology and production efficiency, and the continued use of old knowledge and technology will lead to a decline in competitiveness. Therefore, it is necessity to increase the knowledge-transfer activities between the entities, promote the dissemination of new knowledge, and improve the technology, so as to continuously improve the knowledge reserves and green innovation capabilities of various subjects. After the first phase of development, due to the needs of more stakeholders in the ecological environment, it is necessary to take the enterprise as the core. As the main supplier of the green products market, enterprises can understand the market dynamics and the needs of all of the parties at the same time. Then, the enterprise and the university jointly develop a reasonable solution and appropriate green products. This will promote green innovation performance.

**Figure 4:**
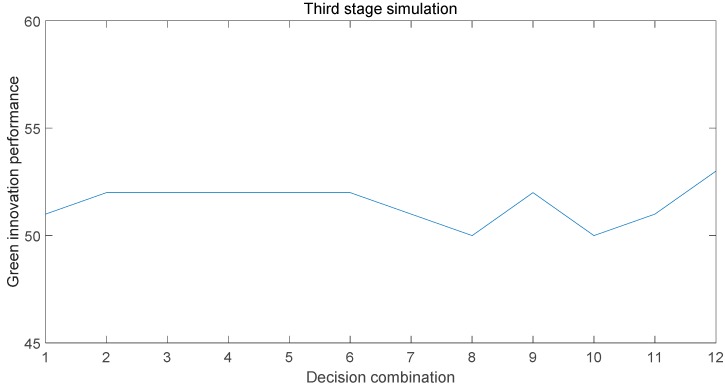
Third-stage simulation results.

After the second simulation, the paper again carries out a third simulation with the second simulation result as the initial state. The simulation results show that when the 12th decision combination is selected, the knowledge transfer performance reaches a relatively optimal value, as shown in Figure 4. It can be seen from the analysis that this can be defined as the mature stage of the development of collaborative innovation centers. The maturity stage is also a period of rapid growth of income. There will be multiple contradictions between the main bodies due to the distribution of interests. Therefore, contract control should be adopted as the major relationship governance mechanism to strengthen cooperation and restraint. At the same time, by improving the efficiency of knowledge and information dissemination, the weak connection relationship can reduce the unfair distribution of internal interests of the Collaborative Innovation Center that can be caused by the strong connection. After cooperation in the early stages, the Collaborative Innovation Center has gradually matured, so both the enterprise and the university should be taken as the core. The dual-center model can promote more convenient and smooth communication between different attribute subjects, increase information transmission efficiency, and improve information symmetry. All of the subjects can grasp the key points of pollution more comprehensively and update the technology from a fundamental level. In the end, the green innovation performance of the collaborative innovation center has been rapidly improved.

Through the simulation of the collaborative innovation center, when the overall arrival of the steady state and the green innovation performance stop improving, the specific values of the steady state can be used as different initial states, and then the combination of strategies is changed to make the green innovation performance continually grow. However, according to the numerical results of performance growth at different stages, it is found that the growth of green innovation performance has a diminishing marginal effect. At the same time, in the process of development, the influence of the 12 different strategies has gradually changed from the initial difference to the same during the development stage. This observation shows that when the collaborative innovation center develops and matures, the green innovation performance will gradually reach the steady state. Even if the decision is replaced, it also can’t produce significant utility. This paper considers this observation to be consistent with life cycle theory. When developing to maturity, the growth value of green innovation performance should be relatively high. If you want to make it rise again, the driving factors should change subversively. This refers to the unanticipated shift in individual or overall factors among all of the factors related to overall innovation performance.

## 5. Conclusions

This paper explores and analyzes the related factors of the Collaborative Innovation Center of Ecological Building Materials and Environmental Protection Equipment in Jiangsu Province by a qualitative simulation method. To solve a large number of related specific problems, all of the relevant influencing factors are compiled through the qualitative analysis method. They are classified as two categories: decision factors and driving factors. We identified the internal interactions between all of the factors. Then, according to the basic content of the QSIM method, this paper makes some improvements to the simulation. Different decision combinations can be selected according to different stage status values to improve the green innovation performance. With the analysis of the simulation results, this paper provides some suggestions for the green innovation performance for the Collaborative Innovation Center of Ecological Building Materials and Environmental Protection Equipment in Jiangsu Province. These suggestions will enable the Collaborative Innovation Center to rapidly develop and improve the comprehensive ability of solving pollution problems, and thus improve the ecological environment and the peaceful development of people and nature. The main contributions of this paper are reflected in two aspects. First, different collaborative centers can get different decision guidance in different state stages. Secondly, for the current research object, the Collaborative Innovation Center can adjust from the relationship governance mechanism, relationship strength, and core leadership, and then improve the performance of green innovation.

There are still some shortcomings in this paper. Firstly, the analysis of the factors affecting the performance of green innovation may not be comprehensive enough. Secondly, the model emphasizes the role of the Collaborative Innovation Center in enhancing the overall development process of green innovation performance. There is no further subdivision of internal factors such as decision combinations. Model characterization needs to be developed at the micro level. Therefore, in future research, an in-depth analysis of the connotation, division of labor, and role of the influencing factors can be carried out, for example, the green innovation capability can be subdivided into technology, knowledge reserve, management ability, and so on. The research can also analyze the whole process of the development of the Collaborative Innovation Center from the perspective of internal heterogeneity and project characteristics, such as a careful study from the perspectives of enterprises, academics, and intermediaries. The optimization and improvement of the simulation algorithm are also the main direction to improve the research results.

## Figures and Tables

**Table 1:** Source of influencing factors.

Influencing Factor	Influencing Factor Description	Author
Green innovation capability	The comprehensive performance of the company’s technology, capital, and experience in green innovation	Hepburn et al. (2018) [18]; Qi et al. (2010) [19]; Lee et al. (2011) [20]; Albort-Morant et al. (2018) [21]; Horowitz et al. (2017) [22]; Du et al. (2017) [23]; Weng, et al. (2015) [24]; Triguero et al. (2013) [25]; Robertson et al. (2018) [26]; Țăpurică et al. (2013) [31]; Boiral et al. (2009) [32]; Wehrmeyer et al. (1995) [33]; Sugita et al. (2015) [34]; Kotter (1990) [35]; Saunila et al. (2018) [36]; Lin et al. (2014) [37]; Ba et al. (2013) [38]; Lioutas et al. (2018) D [39]; Freire (2018) [40]; Kessler et al. (2012) [41]; Huo et al. (2016) [42]; Yongman (2009) [43]; Guo et al. (2018) [44]; Carrion-Flores et al. (2010) [45]; Doran et al. (2016) [46]; Lee (2012) [47].
Green innovation willingness	The willingness to green innovation indicates the enthusiasm of enterprises for green innovation	
Contract norms	The integrity of the contract signed between the various companies in the Collaboration Center	
Credibility	Reputation, cooperation loyalty, etc. of the companies within the collaborative center	
Environmental leadership	Encourage the organization to lead with positive environmental behavior	
Policies	National support for green innovation	
Regional culture	The degree of cultural integration in the region of the center	
Regional environmental pollution	Whether the degree of environmental pollution in the area is serious	
Regional resource utilization	Whether the use of resources in the region is sufficient	
Environmental regulations	Whether the laws and regulations related to environmental issues are perfect	
Green product demand	The level of demand for environmentally-friendly green products	
Green production costs	The manufacturing cost of green products	
Green technology difficulty	The difficulty of research and development of green manufacturing technology	
Interest distribution	Fair and equitable distribution of benefits in the collaborative center	
Communication and coordination	Frequency and effectiveness of communication between enterprises in the collaborative center	
Knowledge transfer	Frequency and effectiveness of knowledge transfer activities	
Stakeholder environmental pressure	Stakeholders’ requirements for the surrounding environment	

**Table 2:** Collaborative innovation network green innovation performance-related factors.

Performance	Innovation Performance	Green Innovation Performance	I_1_
Drivers	Attribute factors	Green innovation capability	I_2_
		Green innovation willingness	I_3_
		Contract norms	I_4_
		Credibility	I_5_
		Environmental leadership	I_6_
	Regional macro factors	Policies	I_7_
		Regional culture	I_8_
		Regional environmental pollution	I_9_
		Regional resource utilization	I_10_
	Regional micro factors	Environmental regulations	I_11_
		Green product demand	I_12_
		Green production costs	I_13_
		Green technology difficulty	I_14_
	Communication factors	Interest distribution	I_15_
		Communication and coordination	I_16_
		Knowledge transfer	I_17_
		Stakeholder environmental pressure	I_18_
Decision	Relationship governance mechanism	Credence	I_19_
		Contract control	I_20_
	Relationship strength	Strong connection	I_21_
		Weak association	I_22_
	Core leadership	Industry	I_23_
		University research	I_24_
		Double center	I_25_

**Table 3:** Driver and performance impact weight table.

Weight	I_1_	I_2_	I_3_	I_4_	I_5_	I_6_	I_7_	I_8_	I_9_	I_10_	I_11_	I_12_	I_13_	I_14_	I_15_	I_16_	I_17_	I_18_
I_1_	0.000	0.118	0.145	0.138	0.026	0.219	0.275	0.277	0.259	0.195	0.128	0.070	0.204	0.207	0.115	0.075	0.086	0.198
I_2_	0.091	0.000	0.038	0.096	0.042	0.166	0.000	0.000	0.137	0.137	0.000	0.000	0.151	0.152	0.159	0.119	0.172	0.104
I_3_	0.130	0.161	0.000	0.138	0.067	0.085	0.000	0.000	0.049	0.049	0.000	0.026	0.000	0.000	0.031	0.046	0.056	0.071
I_4_	0.026	0.038	0.101	0.000	0.042	0.085	0.000	0.037	0.022	0.022	0.000	0.000	0.000	0.000	0.019	0.075	0.022	0.045
I_5_	0.017	0.038	0.025	0.024	0.000	0.055	0.029	0.037	0.000	0.000	0.039	0.000	0.017	0.000	0.212	0.119	0.086	0.027
I_6_	0.012	0.022	0.011	0.042	0.067	0.000	0.000	0.037	0.000	0.000	0.039	0.103	0.000	0.000	0.051	0.046	0.035	0.045
I_7_	0.042	0.000	0.016	0.000	0.017	0.021	0.000	0.067	0.016	0.016	0.340	0.026	0.026	0.000	0.031	0.029	0.035	0.017
I_8_	0.026	0.022	0.016	0.024	0.067	0.085	0.043	0.000	0.137	0.137	0.039	0.189	0.017	0.000	0.051	0.075	0.035	0.027
I_9_	0.017	0.022	0.145	0.193	0.017	0.021	0.383	0.157	0.200	0.20	0.340	0.249	0.113	0.112	0.000	0.000	0.022	0.147
I_10_	0.091	0.038	0.038	0.042	0.026	0.055	0.086	0.108	0.000	0.000	0.000	0.026	0.040	0.036	0.019	0.019	0.035	0.045
I_11_	0.062	0.022	0.072	0.042	0.204	0.000	0.029	0.067	0.034	0.034	0.000	0.044	0.026	0.000	0.019	0.019	0.012	0.017
I_12_	0.026	0.060	0.145	0.042	0.017	0.021	0.000	0.037	0.022	0.022	0.039	0.000	0.060	0.036	0.031	0.019	0.056	0.012
I_13_	0.130	0.060	0.101	0.024	0.067	0.021	0.000	0.000	0.069	0.069	0.000	0.044	0.000	0.036	0.079	0.046	0.022	0.104
I_14_	0.182	0.118	0.016	0.024	0.107	0.021	0.155	0.000	0.096	0.096	0.000	0.103	0.204	0.000	0.051	0.175	0.172	0.045
I_15_	0.062	0.022	0.072	0.024	0.067	0.055	0.000	0.067	0.000	0.000	0.000	0.026	0.060	0.023	0.000	0.046	0.056	0.024
I_16_	0.026	0.022	0.016	0.024	0.042	0.035	0.000	0.037	0.000	0.000	0.000	0.026	0.017	0.036	0.079	0.000	0.086	0.045
I_17_	0.042	0.215	0.016	0.024	0.017	0.021	0.000	0.037	0.022	0.022	0.000	0.026	0.040	0.361	0.031	0.075	0.000	0.024
I_18_	0.017	0.022	0.025	0.096	0.107	0.035	0.000	0.037	0.000	0.000	0.000	0.044	0.026	0.000	0.019	0.019	0.012	0.000

**Table 4:** Weighted table of decisions on drivers and performance.

Weight	I_2_	I_3_	I_4_	I_5_	I_6_	I_7_	I_8_	I_9_	I_10_	I_11_	I_12_	I_13_	I_14_	I_15_	I_16_	I_17_	I_18_
I_19_	0.833	0.889	0.50	0.143	0.875	0.500	0.667	0.167	0.250	0.200	0.250	0.333	0.333	0.667	0.857	0.667	0.857
I_20_	0.167	0.111	0.50	0.857	0.125	0.500	0.333	0.833	0.750	0.800	0.750	0.667	0.667	0.333	0.143	0.333	0.143
I_21_	0.250	0.857	0.80	0.250	0.857	0.500	0.500	0.250	0.333	0.250	0.250	0.200	0.250	0.667	0.857	0.250	0.750
I_22_	0.750	0.143	0.200	0.750	0.143	0.500	0.500	0.750	0.667	0.750	0.750	0.800	0.750	0.333	0.143	0.750	0.250
I_23_	0.078	0.070	0.724	0.200	0.623	0.200	0.200	0.124	0.114	0.557	0.623	0.539	0.093	0.200	0.320	0.083	0.297
I_24_	0.068	0.723	0.083	0.200	0.239	0.200	0.200	0.663	0.323	0.123	0.137	0.297	0.685	0.200	0.123	0.193	0.164
I_25_	0.234	0.206	0.193	0.600	0.137	0.600	0.600	0.213	0.563	0.320	0.239	0.164	0.221	0.600	0.557	0.724	0.539

**Table 5:** Staged driver, optimal decision combination, optimal green innovation performance.

Stage	I_1_	I_2_	I_3_	I_4_	I_5_	I_6_	I_7_	I_8_	I_9_	I_10_	I_11_	I_12_	I_13_	I_14_	I_15_	I_16_	I_17_	Optimal Decision Combination	Optimal Green Innovation Performance
First	10	13	15	12	8	50	6	93	7	10	50	92	96	11	10	10	90	*X*_1_, *Y*_1_, *Z*_2_	37
Second	40	39	41	36	52	61	47	72	49	14	57	72	77	53	46	43	60	*X*_1_, *Y*_2_, *Z*_1_	49
Third	64	63	65	42	80	76	72	60	65	13	57	62	72	75	73	66	28	*X*_2_, *Y*_2_, *Z*_3_	53
